# Cyclophosphamide Pharmacogenomic Variation in Cancer Treatment and Its Effect on Bioactivation and Pharmacokinetics

**DOI:** 10.1155/2024/4862706

**Published:** 2024-06-27

**Authors:** Ibrahim El-Serafi, Sinclair Steele

**Affiliations:** ^1^ Basic Medical Sciences Department College of Medicine Ajman University, Ajman, UAE; ^2^ Department of Hand Surgery, and Plastic Surgery and Burns Linköping University Hospital, Linkoöping, Sweden; ^3^ Pathological Sciences Department College of Medicine Ajman University, Ajman, UAE

## Abstract

Cyclophosphamide (Cy) is a prodrug that is mainly bioactivated by cytochrome P450 (CYP) 2B6 enzyme. Several other enzymes are also involved in its bioactivation and affect its kinetics. Previous studies have shown the effect of the enzymes' genetic polymorphisms on Cy kinetics and its clinical outcome. These results were controversial primarily because of the involvement of several interacting enzymes in the Cy metabolic pathway, which can also be affected by several clinical factors as well as other drug interactions. In this review article, we present the effect of CYP2B6 polymorphisms on Cy kinetics since it is the main bioactivating enzyme, as well as discussing all previously reported enzymes and clinical factors that can alter Cy efficacy. Additionally, we present explanations for key Cy side effects related to the nature and site of its bioactivation. Finally, we discuss the role of busulphan in conditioning regimens in the Cy metabolic pathway as a clinical example of drug-drug interactions involving several enzymes. By the end of this article, our aim is to have provided a comprehensive summary of Cy pharmacogenomics and the effect on its kinetics. The utility of these findings in the development of new strategies for Cy personalized patient dose adjustment will aid in the future optimization of patient specific Cy dosages and ultimately in improving clinical outcomes. In conclusion, CYP2B6 and several other enzyme polymorphisms can alter Cy kinetics and consequently the clinical outcomes. However, the precise quantification of Cy kinetics in any individual patient is complex as it is clearly under multifactorial genetic control. Additionally, other clinical factors such as the patient's age, diagnosis, concomitant medications, and clinical status should also be considered.

## 1. Introduction

Cancers are a group of diseases which are characterized by disordered and continuous cell growth. Humanity has been dealing with the blight of cancer as early as the ancient Egyptian era; essentially, our medical skills have been unable to satisfactorily manage cancer for thousands of years [1].

Cancers can be divided into solid tumors, such as breast cancer and ovarian cancer, and hematological malignancies [2] including lymphoma, myeloma, and leukemia [3]. Surgery has been the first choice for cancer treatment for many centuries [1]. However, none of the available treatments alone, i.e., surgery, chemotherapy, or radiotherapy, can cure all patients. Therefore, the combination of surgery and cytostatics and/or radiation has become the standard treatment for several cancer types [1]. However, for disseminated disease involving several organs, such as metastatic solid cancer and hematological malignancies, chemotherapy remains the dominant treatment. Regrettably, for some patients, neoplastic recurrence or primary treatment resistant disease inhibits potentially curative approaches. Consequently, such patients may be offered hematopoietic stem cell transplantation (HSCT).

## 2. Purpose of the Review

Cyclophosphamide (Cy) is one of the most important cytostatics used for the treatment of several types of malignancies as well as for the conditioning prior to HSCT. It is a prodrug that is activated by several enzymes. Additionally, other important enzymes are involved in its metabolic pathway. The effect of these enzymes' genetic polymorphisms on Cy kinetics and its clinical outcomes is often contradictory and unclear. Moreover, other clinical factors may impact Cy kinetics. In this review article, we summarize the effect of different enzymes' polymorphisms on Cy kinetics and we present explanations for key Cy side effects. We also discuss the busulphan (Bu)/Cy conditioning regimen as a commonly used combination that is a clinical example of drug-drug interactions involving several polymorphic enzymes.

## 3. Background

### 3.1. Cytostatics

The classification of a cytostatic medication depends on its mechanism of action. Accordingly, these drugs may be alkylating agents, antimetabolites, antitumor antibiotics, mitosis inhibitors, topoisomerase inhibitors, enzymes, or hormonal agents.

Alkylating agents are the oldest class of drugs used in cancer treatment. They act by attaching an alkyl group to the guanine base of DNA at the imidazole ring and forming covalent bonds with amino, phosphate, and carboxyl groups. Such chemical damage can trigger apoptosis. Alkylating agents include nitrogen mustard, chlorambucil, melphalan, cyclophosphamide, ifosfamide, thiotepa, busulphan, treosulfan, and hexamethylmelamine. They are commonly used in conditioning prior to HSCT as well as in the treatment of hematological malignancies and solid tumors such as breast adenocarcinomas, lung carcinomas, and ovarian adenocarcinomas.

### 3.2. Cyclophosphamide

Cyclophosphamide is an alkylating agent that has been marketed since 1959 and is considered to be one of the most important drugs in cancer therapy. It is used at high doses as a part of the conditioning regimen prior to HSCT, either in combination with other cytostatics or with TBI [4–8]. Cy is also used at lower doses post-HSCT to prevent graft rejection and to decrease the severity of GVHD [9–13]. Additionally, Cy is widely used in lower doses for the treatment of hematological malignancies as well as solid tumors including ovarian adenocarcinomas, breast adenocarcinomas, small cell lung carcinomas, pancreatic ductal adenocarcinomas, and neuroblastomas in both adults and children [14–24]. Moreover, Cy is also a potent immunosuppressive agent that affects both T- and B-lymphocytes as it is capable of attenuating both humoral and cell-mediated immune responses [25]. Due to these immunosuppressive effects, low doses of Cy are used in the treatment of several autoimmune diseases such as glomerulonephritis, multiple sclerosis, rheumatoid arthritis, systemic lupus erythematosus, and Sjögren´s syndrome as well as in the preconditioning of hosts to prevent organ transplant rejection [26–29].

**Figure 1:**
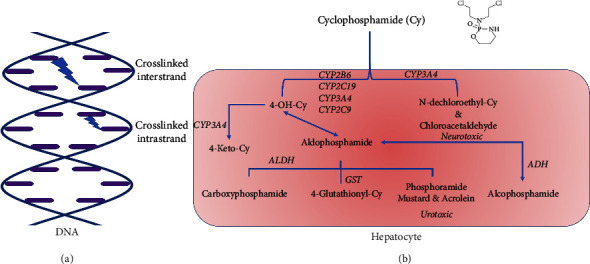
Cyclophosphamide mechanism of action on DNA showing the inter- and intrastrand crosslinks that cause DNA damage and trigger apoptosis in cancer cells (a) and its metabolic pathway showing its bioactivation from a prodrug to an active agent and its terminal cytotoxic metabolites (b).

As an alkylating agent, Cy covalently binds to the guanine-N-7 base of DNA and produces DNA damage that triggers apoptosis when the cellular machinery fails in its reparative role (Figure 1(a)). It is a prodrug that has to be bioactivated to exert its effect. Alkylating agents share the Cy mechanism of action, as well as common cyclophosphamide drug-drug interactions are listed in Table 1.

### 3.3. Hematopoietic Stem Cell Transplantation

HSCT is a curative treatment for malignancies such as leukemia, lymphomas, and some solid tumors as well as for nonmalignancies including metabolic disorders and aplastic anemia [30]. However, several acute and chronic complications such as graft-versus-host disease (GVHD), infections, and sinusoidal obstruction syndrome (SOS) are known to occur and may alter the clinical outcome.

**Figure 2:**
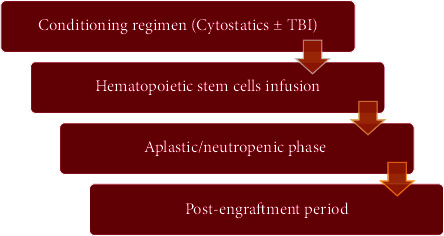
Hematopoietic stem cell transplantation phases. TBI: total body irradiation.

Hematopoietic stem cells may be harvested from bone marrow, peripheral blood, or umbilical cord blood. An HSCT is performed via four main phases starting with the conditioning regimen, which aims to eliminate the malignant cells, provides a space for the donor cells, and provides enough immune suppression of the host immune system to avoid graft rejection. After conditioning, the patient receives the hematopoietic stem cells. Within a few weeks, the patient experiences the aplastic/neutropenic phase which is then followed by the postengraftment period (Figure 2).

The conditioning regimen is one of the most important steps in HSCT. Conditioning regimens can be divided into either total body irradiation (TBI)-based TBI and cytostatics or chemotherapy-based combinations of cytostatics without TBI [31].

Radiotherapy was first used as the sole conditioning regimen. However, it was associated with many acute and chronic complications such as stomatitis, enteritis, infection-related death, and secondary leukemia post-transplantation [32]. Additionally, TBI has also been associated with late complications such as secondary malignancies, CNS damage, impaired growth, and endocrine dysfunction [33].

Development of cytostatic agents contributed to the use of cytostatics in conditioning regimens and reduced the dosage of radiotherapy. Cy was first added to TBI; however, subsequent studies in children have shown growth impairment as a late side effect [34, 35]. Later on, Bu was added with Cy, instead of TBI, since Bu has less severe delayed effects than TBI [32, 36]. The Bu/Cy regimen proved to be as good as TBI-based regimens, especially in patients with myeloid leukemia.

Nowadays, many other cytostatics are also used for conditioning prior to HSCT such as fludarabine and treosulfan [37]. Treosulfan is considered to be a less toxic alternative to Bu with a reduced incidence of acute GVHD and improved overall survival [38]. The conditioning regimen is selected with appropriate regard to the diagnosis, disease stage, patient age, patient heath status, comorbidities, and risk of transplantation-related complications.

## 4. Cyclophosphamide Metabolism

Cyclophosphamide is a prodrug that is metabolized mainly in the liver by cytochrome P450 enzymes (CYPs) into several metabolites. However, the predominant and active metabolite is 4-hydroxy-cyclophosphamide (4-OH-Cy) which corresponds to more than 80% of the total dose of Cy [39]. Most Cy terminal metabolites are cytotoxic substances such as 4-OH-Cy, which is degraded to phosphoramide mustard (PM) and acrolein that is responsible for urotoxicity (hemorrhagic cystitis) [40]. PM alkylates the guanine base of DNA at the *N*7 position of the imidazole ring and then triggers apoptosis due to the formation of guanine-adenine intrastrand crosslinks [26]. An alternative metabolic pathway involves the *N-*dechloroethylation of cyclophosphamide which results in an inactive metabolite, *N*-dechloroethyl-Cy, and a neurotoxic metabolite, chloroacetaldehyde (CA) (Figure 1(b)) [41–44].

### 4.1. Cytochrome P450

Cytostatics are foreign substances or xenobiotics that are metabolized by different enzymes in order to be excreted from the body. The main aim of these reactions is to make the drugs more hydrophilic and to facilitate their excretion in an aqueous medium. Most of these reactions occur successfully in the liver; however, in spite of these reactions, some drugs may be excreted unchanged.

The metabolism of cytostatics occurs in two phases. In phase I, the major reaction involved is hydroxylation, which is catalyzed mainly by CYPs. Other reactions include desulfuration, deamination, dehalogenation, epoxidation, peroxygenation, and reduction. During this phase, most of the drugs become pharmacologically less active or pharmacologically completely inactive. However, in contrast, some drugs such as cyclophosphamide are converted from an inactive prodrug to a biologically active metabolite [45].

In phase II, the compounds produced in phase I reactions, or compounds that already possess polar substituents, are converted by specific enzymes to more polar metabolites through further conjugation with polar molecules such as glutathione, glucuronic acid, and/or sulfate [45].

Cytochrome P450 enzymes are a superfamily that are mostly membrane-associated hemoproteins that can be found in the inner membrane of mitochondria or in the endoplasmic reticulum of the cells. CYPs are present in many organs, with the greatest quantity found in the liver and the small intestine. They are involved in the metabolism of many drugs and xenobiotics [46].

Most of the CYPs are inducible, which is mainly due to an increase in mRNA transcription. The administration of drugs such as phenobarbital can cause hypertrophy of the smooth endoplasmic reticulum and an increase in the amount of CYP production within a few days. Some cases of induction involve stabilization of mRNA, enzyme stabilization, or other mechanisms that affect protein translation. In contrast to the inducibility of CYPs, specific drugs can also inhibit CYP activities [47, 48].

The most common reactions catalyzed by CYPs are monooxygenase reactions. In this type of reaction, one atom of oxygen is inserted into an organic substrate, while the other oxygen atom is reduced to water:

(1) $$\begin{array}{c} \text{RH}+O_{2}+\text{NADPH}+H^{+}⟶\text{ROH}+H_{2}O+{\text{NADP}}^{+}. \end{array}$$

CYP Phase I reactions include the metabolism of xenobiotics such as particular medicines, carcinogens, pesticides, and pollutants as well as several endogenous compounds such as steroids and fatty acids [46].

In humans, there are 57 genes and more than 58 pseudogenes for CYP production and these are divided into 18 families and 43 subfamilies [49]. Many CYPs exist in polymorphic forms that differ in their catalytic activity. This is believed to explain the interindividual variations in drug response in a given population. The most common enzymes reported to be involved in drug metabolism are CYP1A1, CYP1A2, CYP2A6, CYP2B6, CYP2C8, CYP2C9, CYP2C19, CYP2D6, CYP2E1, CYP3A4, and CYP4A11 [48, 50, 51].

Cytochrome P450 oxidoreductase (POR) is the main enzyme responsible for the electron transfer from NADPH to CYP. POR is important in the metabolism of drugs and xenobiotics, and its allelic variants and variability in expression can have clinical implications [52, 53]. Cytochrome *b*_5_ is another hemoprotein that has the same function as POR.

### 4.2. CYP2B6 and POR Polymorphisms

Genetic polymorphism has been associated with variable levels of gene expression in the liver. Several CYP polymorphisms have been identified that possess different expression levels and hence different activities. In general, the first identified polymorphism is always known as ^*∗*^*1* and is considered as the standard with respect to comparison with the other polymorphisms. In many cases, the ^*∗*^*1* polymorphism may not be the most common version in humans.

CYP2B6 is the main enzyme in Cy bioactivation and is one of the common enzymes whose expression level as well as its activity is affected by genetic polymorphism [41, 54]. Other enzymes such as CYP2J2, CYP3A4, CYP3A5, CYP2C9, and CYP2C19 are also involved in Cy metabolism [55–62].

The gene for CYP2B6 is located in the middle of chromosome 19 [63] and is mainly expressed in the liver; however, it has been detected in extrahepatic tissues such as the intestines, kidneys, brain, lungs, and skin [64–67]. CYP2B6 has been reported to be involved in the metabolism of many other drugs including ifosfamide, diazepam, and efavirenz as well as in the synthesis of endogenous compounds such as cholesterol and steroids [65, 68–70].

Several studies have shown great interindividual variation in expression and catalytic activity of CYP2B6 which may also be attributed to the genetic polymorphism of this enzyme [71–73]. Human CYP2B6 expression levels for the C1459T mutation (alleles ^*∗*^*5* and ^*∗*^*7*) are common and known to be significantly lower than for *CYP2B6*^*∗*^*1* [65]. In Caucasians, the SNP frequencies of A785G and G516T are 33% and 29%, respectively [65]. These SNPs are present in several *CYP2B6* allelic variants such as *2B6*^*∗*^*4*, *2B6*^*∗*^*6*, *2B6*^*∗*^*7*, and *2B6*^*∗*^*9*. In the population of Pakistan, lesser allelic frequencies of 33.8% for *CYP2B6*^*∗*^*6*, 25.8% for *CYP2B6*^*∗*^*4*, and 6.5% for *CYP2B6*^*∗*^*3* have been observed, whereas the wild-type genotype frequencies are 48.57% for *CYP2B6*^*∗*^*6*, 59.79% for *CYP2B6*^*∗*^*4*, and 90.20% for *CYP2B6*^*∗*^*3*, indicating a significant prevalence of poor metabolizers of CYP2B6, especially the ^*∗*^*6* variant [74]. *CYP2B6* polymorphism has been reported to affect the kinetics of several drugs. For example, after the same administered dose, the mirtazapine drug concentration was higher in patients with the *CYP2B6*^*∗*^*6/*^*∗*^*6 variant* [75]. Furthermore, the frequencies of different alleles vary in different populations. For example, Ribaudo et al. have shown that *CYP2B6*^*∗*^*9* is more frequent amongst African-American individuals compared to Caucasian-American individuals, which resulted in a two-fold longer plasma half-life of efavirenz in homozygotes for this allele [76]. In total, more than fifty different alleles containing point mutations have been identified. Generally, most of these mutations are silent; however, five of them result in amino-acid substitutions in exons 1, 4, 5, and 9 [65].

As mentioned previously, POR is the main electron donor for all microsomal CYP monooxygenases. Accordingly, POR deficiency may lead to serious complications such as disordered steroidogenesis, abnormal genitalia, bone abnormalities, and William's syndrome [77–79]. POR polymorphisms have been shown to affect CYP-mediated drug metabolism as well as causing direct bioactivation of prodrugs [80]. POR polymorphisms affect the amount of enzyme produced which may affect the activities of cytochrome P450 enzymes such as CYP1A2, 2C9, 2C19, 2D6, 3A4, and 3A5 [81–84], especially for compounds which are rapidly metabolized and where the electron transfer from NADPH via POR can be rate-limiting. Our literature review indicates that *POR*^*∗*^*28* is the only polymorphism reported to increase CYP activity *in vivo* [85]. Many other polymorphisms are known to decrease its activity *in vivo*, particularly *POR*^*∗*^*2, 3, 4*, and *5*.

Chen et al. studied the effect of the POR genotype on CYP2B6 bupropion metabolism. Their results showed up to a 74% reduction in CYP2B6 activity with certain *POR* polymorphisms *in vitro* [86]. Subsequent research confirmed these findings and reported that *S*-mephenytoin *N*-demethylation by CYP2B6 varied with the specific POR polymorphism present in human livers [86, 87].

POR variability also affects CYPs other than CYP2B6, such as CYP2C9 activity when incubated with flurbiprofen, diclofenac, and/or tolbutamide. These drugs, like Cy, are metabolized rapidly [83]. The effect of POR variants and their expression levels varies with the substrate and the CYP enzyme variant; for example, *POR* polymorphic variants A287P and/or R457H are associated with no detectable CYP2D6 metabolism of 7-ethoxymethoxy-3-cyanocoumarin (EOMCC), while the Q153R polymorphism increased CYP2D6 activity against EOMCC *in vitro* [82].

Steroidogenic activity is dependent mainly on CYP1A2 and CYP2C19. POR variants affected the activities of these enzymes to different extents. *POR* polymorphisms A287P and/or R457H have reduced CYP1A2 and CYP2C19 catalytic activities against EOMCC. The A503V polymorphism demonstrated 85% of the wild-type activity with CYP1A2 and 113% of the wild-type activity with CYP2C19, while Q153R polymorphism increased both CYP1A2 and CYP2C19 activities [81].

CYP3A4 completely lost its ability to metabolize testosterone *in vitro* in two of the *POR* polymorphisms, Y181D and A287P. Other *POR* polymorphisms, such as K49N, A115V, and G413S, resulted in an up to 65% increase in CYP3A4 activity on a testosterone substrate [86]. Moreover, tacrolimus is metabolized mainly by CYP3A5; in individuals with *POR*^*∗*^*28* polymorphism, tacrolimus exposure was significantly decreased [84].

A study of human liver samples showed that four *POR* polymorphisms (K49N, L420M, A503V, and L577P) were associated with reductions in both POR and in drug-metabolizing CYP activities. The same study also showed intronic polymorphisms that altered POR activity [88].

### 4.3. The Effect of CYPs and POR Polymorphisms and Their Expression Levels on Cyclophosphamide Pharmacokinetics

Several studies have investigated the clinical efficacy of Cy alone or in combination with other cytostatics or radiotherapy [5, 89] Studies on Cy kinetics have shown a high interindividual variation in elimination half-life and clearance which then affects treatment efficacy and toxicity [41, 44, 90]. This variation may be explained by polymorphisms in *CYP2B6*, the main enzyme responsible for the conversion of Cy to its active form, 4-OH-Cy in the liver [44, 54, 71–73, 91–93].

This variability has been attributed to polymorphisms in the *CYP2B6* gene, such as *CYP2B6*^*∗*^*4*, *CYP2B6*^*∗*^*6*, *CYP2B6*^*∗*^*7*, and *CYP2B6*^*∗*^*9* [57, 73, 94–97]. Common alleles were reported to affect Cy kinetics by either decreased liver protein expression or altered function of the enzyme. These alleles include *CYP2B6*^*∗*^*2* (C64T, R22C), *CYP2B6*^*∗*^*4* (A785G, S262R), *CYP2B6*^*∗*^*5* (C1459T, R487C), *CYP2B6*^*∗*^*6* (G516T, Q172H and A785G, K262R), *CYP2B6*^*∗*^*7* (G516T, Q172H; A785G, K262R; and C1459T, R487C), and *CYP2B6*^*∗*^*9* (Q172H) [65, 95, 98–101]. *CYP2B6rs2279343* was reported to be associated with improved survival of pediatric rhabdomyosarcoma Cy treated patients [102]. Barnett et al. suggested that the effect of CYP2B6 and CYP2C9 on Cy clearance is more significant in children less than two years of age, as compared to older children [103]. In addition, some more rare SNPs have been reported to result in absent or nonfunctional enzymes [104].

On the other hand, other studies have concluded that CYP polymorphisms (including CYP2B6) do not play a significant role in the response variation to Cy with respect to complete remission, while clinical factors such as patients' ages and cancer grades may be more significant [54–56, 105–109]. Recently, a lack of association of CYP2B6 pharmacogenetics with Cy toxicity in patients was reported; moreover, aldehyde dehydrogenases 1A1 (ALDH1A1) rs8187996 may have a lower risk of Cy toxicity compared to wild-type patients [110]. In pediatric patients with non-Hodgkin's B-cell lymphomas, the CYP2B6 genotype was reported to influence Cy but had no clear impact on the clinical outcome [111].

As mentioned previously, other enzymes such as CYP3A4, CYP3A5, CYP2C9, CYP2C19, and CYP2J2 were reported to be involved in Cy bioactivation but to a lesser extent [18, 51, 55, 56, 59, 60, 92, 100, 101, 112, 113]. Cy-treated patients with the variant CYP2C9 had an increased risk of leukopenia but responded better to treatment compared to those who were carriers of wild-type CYP2C9 [114].

The expression levels of C1459T mutation (alleles ^*∗*^*5* and ^*∗*^*7*) of CYP2B6 are significantly lower than for *CYP2B6*^*∗*^*1* [65], and this leads to a higher Cy intrinsic clearance both *in vitro* and *in vivo* for the latter [90, 95]. Additionally, a higher activity of *CYP2B6*^*∗*^*6* was reported in Cy bioactivation in comparison with a decreased activity of efavirenz bioactivation. In this *in vitro* study, the tested microsomes contained CYP2B6.1, CYP2B6.4, or CYP2B6.6 but with the same POR activity and POR/CYP ratio in each batch [99].

**Figure 3:**
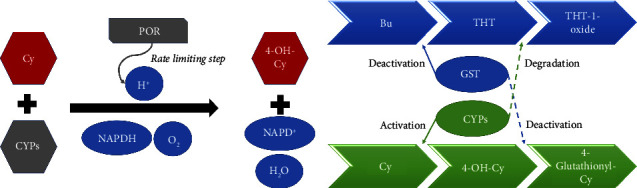
Possible causes of variation in cyclophosphamide kinetics. (a) The different POR enzyme polymorphisms and expression levels that can affect the drug bioactivation. (b) Sequence of administration - cyclophosphamide administration after busulphan since several enzymes are involved in both drugs' metabolic pathways. Bu: busulphan; Cy: cyclophosphamide; CYP: cytochrome P450; GST: glutathione S-transferase; POR: cytochrome P450 oxidoreductase; THT: tetrahydrothiophene.

The formation of 4-OH-Cy is a fast reaction which means that the rate-limiting step may be the electron transfer from the POR to CYP [88] (Figure 3(a)). In an *in vitro* experiment with recombinant human CYP2B6.1, the intrinsic clearance of Cy was clearly proportional to the POR/CYP ratio despite the *K*_*m*_ being almost constant in all batches, confirming a good positive linear correlation between Cy clearance and the POR/CYP ratio [115]. Additionally, results obtained from *POR* gene expression measurements in patients conditioned with Cy showed significant *POR* up-regulation after Cy infusion, with high interindividual variations in gene expression. Still, *POR* expression showed a significant positive correlation with the concentration ratio 4-OH-Cy/Cy. In those patients, some were carriers for *POR*^*∗*^*28*; however, others also had high *POR* expression, possibly due to other *POR* polymorphisms not yet described or effects on nuclear receptors or other factors involved in *POR* regulation, also resulting in higher inducibility [115]. In summary, (i) CYPs are therapeutically important and (ii) polymorphisms affecting *POR* expression or activity should be considered for dose adjustment in order to achieve optimal therapeutic drug plasma concentrations. The most important enzymes involved in the cyclophosphamide metabolic pathway and the most common polymorphisms affecting its kinetics are listed in Table 2.

### 4.4. The Role of CYP2J2 in Cyclophosphamide Extrahepatic Bioactivation

CYP2J2 is another CYP involved in the metabolism of xenobiotics. It is encoded by the *CYP2J2* gene which has been mapped to the short arm of chromosome 1 in humans and chromosome 4 in mice [116].

CYP2J2 is active mainly in the gastrointestinal and cardiovascular systems [117–119]. CYP2J2 has been reported to metabolize several drugs, particularly in extrahepatic tissues [119–121]. High CYP2J2 activity in the intestines could contribute to the first-pass metabolism of some drugs [120–122]. Moreover, CYP2J2 is dominant in the presystemic elimination of astemizole in human and rabbit small intestines [121].

In the human heart, CYP2J2 is responsible for the epoxidation of endogenous arachidonic acid to four regioisomeric epoxyeicosatrienoic acids (EETs) released in response to certain stimuli such as ischemia [123]. Transgenic mice overexpressing *CYP2J2* have been shown to have less extensive infarctions and more complete recovery after ischemia [124–126]. These mice were also better protected against global cerebral ischemia associated with increased regional cerebral blood flow [127].

Additionally, CYP2J2 has been also associated with malignancy as it is highly expressed in cells of hematological and solid tumors [128–135]. Chen et al. have reported that *CYP2J2* is highly expressed in human- and mouse-derived malignant hematological cell lines (K562, HL-60, Raji, MOLT-4, SP2/0, Jurkat, and EL4 cells) as well as in peripheral blood and bone marrow cells of leukemic patients [130]. In these patients, a high level of expression of *CYP2J2* was associated with accelerated neoplastic proliferation and attenuated apoptosis. *CYP2J2* overexpression also enhanced malignant xenograft growth [130].

*CYP2J2* is also overexpressed in ovarian cancer and lung cancer metastases [131, 133], and its inhibition by terfenadine-related compounds has been shown to suppress the proliferation of human cancer cells both *in vitro* and *in vivo,* which implies that CYP2J2 expression is part of a protective mechanism for cancer cell survival. *CYP2J2* inhibition has been shown to suppress the proliferation of cancer cells [129]. The expression of *CYP2J2* in HL-60 cells can account for the Cy cytotoxic effects in these cells [136]. HL-60 cells predominantly express CYP1A1 and CYP1B1 but not CYP2B6; however, neither was previously reported to be involved in Cy bioactivation [136, 137].

Interestingly, CYP2J2 has been reported to be involved in the bioactivation of Cy [58, 59]. A previous study has shown that Cy treatment upregulated the expression of *CYP2J2*; however, the interindividual variation was substantial. For example, Cy-treated patients who were carriers of *CYP2J2* SNP “rs1056596” (A/T) demonstrated low *CYP2J2* expression [59]. Despite the high variability, the expression of CYP2J2 was significantly correlated with the bioactivation of Cy as expressed by the concentration ratio of 4-OH-Cy/Cy [59, 138]. In addition, the inhibition of CYP2J2 in HL-60 cells reduced 4-OH-Cy formation and concomitantly increased the cell viability, which supports the role of CYP2J2 in Cy bioactivation [130]. In the Ethiopian population, it was reported that patients carrying the *CYP2J2*^*∗*^*7* allele with low baseline blood counts were at a higher risk for chemotherapy-induced hematologic toxicities [58]. In some populations, the ratio *V*_max_*/K*_*m*_ (*V*_max_ is the maximum velocity of the reaction where all the enzymes are saturated with the substrate, while *K*_*m*_ is the concentration of the substrate at which half of the maximum velocity is achieved) for CYP2J2 was higher compared to that obtained for CYP2B6 [139]. This suggests that CYP2J2 may be the enzyme predominantly responsible for Cy bioactivation in these patients and not CYP2B6 as described earlier [7, 10].

The abovementioned reports underline the importance of CYP2J2's role in Cy bioactivation and its involvement in drug-related toxicities, especially in organ specific toxicities where *CYP2J2* is highly expressed. The urinary bladder, heart, and intestines are subject to these considerations. Hemorrhagic cystitis as well as the alteration of intestinal permeability accompanying diarrhea (as a marker of intestinal barrier dysfunction) were common side effects that were observed following treatment with Cy in several patients [40, 139–141]. High doses of Cy were reported to be correlated with acute cardiac toxicity resulting in heart failure or a decrease in systolic function [142]. In addition, it has also been shown that damage to the endothelial cells in the mesenteric artery was correlated with Cy treatment [143, 144].

Finally, drugs metabolized via CYP2J2 and concomitantly used during Cy conditioning may alter Cy kinetics and hence the treatment efficacy and increase the Cy-related toxicities. This may explain some of the major side effects reported after HSCT.

### 4.5. Glutathione Importance in Cyclophosphamide Metabolism

Glutathione (GSH) is a nonessential tripeptide that has an unusual peptide linkage between the amino group of cysteine and the carboxyl group of the glutamate side chain. The sulfhydryl (thiol) group (SH) of cysteine serves as a proton donor and is responsible for the biological activity of glutathione.

Glutathione exists in reduced and oxidized states. In the reduced state, the thiol group of cysteine is able to donate a reducing equivalent (H^+^ + e^−^) to other unstable molecules. This reaction converts the glutathione into its active form that can react with another reactive glutathione to form a dimer glutathione disulfide (GSSG). It is important to have a high concentration of glutathione in cells for this reaction to occur. The reaction is often catalyzed by glutathione S-transferases (GSTs) which are present mainly in the cytosol of liver cells. GSH can be regenerated from GSSG by the enzyme glutathione reductase [145].

GSH is the major endogenous antioxidant. Additionally, it has an important role in the conjugation of drugs and other xenobiotics as well as in maintaining exogenous antioxidants such as vitamins C and E in their reduced (active) forms. GSH also plays a major role in several biochemical reactions such as DNA synthesis and repair, protein synthesis, amino-acid transport, and enzyme activation [146].

GSH is very important for Cy metabolism. As mentioned previously, Cy end-metabolites such as acrolein and PM are cytotoxic. These metabolites are responsible for the urotoxicity and cardiotoxicity reported in patients treated with Cy [41–44]. GSH reacts with the active metabolite, 4-OH-Cy, as well as its toxic metabolite, acrolein [147, 148]. Acrolein is highly toxic when administered to perfused rat hearts, isolated coronary blood vessels, or incubated with cardiac myocytes and isolated cardiac mitochondria [149–151].

In Cy treated patients, acrolein is metabolized and detoxified by GSTP via conjugation with GSH. In the absence of GSH, acrolein reacts with circulating and cardiac proteins to form protein-acrolein adducts that may contribute to cardiac injury and cardiotoxicity [148]. Such knowledge can be very valuable for patients with certain *GSTP1* polymorphisms that were reported to alter function and increases the risk of Cy-toxicities [152–156].

Additionally, GSTP was reported as a protective agent against endothelial dysfunction as well as Cy-induced urotoxicity and cardiotoxicity [62, 148, 157, 158].

### 4.6. The Importance of Epigenetics in Drug Metabolism

Recently, epigenetic mechanisms have been reported to be important in drug treatment [159]. Epigenetic modifiers contribute to the interindividual variations in drug metabolism. A novel class of drugs, termed epidrugs, has been reported from clinical trials to intervene in the epigenetic control of gene expression. Furthermore, epigenetic biomarkers can be used in monitoring patients' disease prognosis and treatment.

## 5. Busulphan/Cyclophosphamide as a Common Conditioning Regimen

Busulphan/cyclophosphamide has been one of the most commonly used conditioning regimens in the clinical setting in recent decades [160]. Busulphan is predominantly metabolized via conjugation with GSH [161–164] that is catalyzed by GSTA1 [112, 165, 166]. The conjugation of Bu with glutathione results in the formation of a sulfonium ion which is unstable and decomposes to tetrahydrothiophene (THT) and an N-acetyl-cysteine sulfonium ion. THT is oxidized to THT 1-oxide that is further oxidized to sulfolane and finally to 3-hydroxy sulfolan [161–164]. Other hepatic enzymes, including flavin-containing mono-oxygenase-3 (FMO3) and CYPs, are involved in Bu metabolism and affect its kinetics [167, 168]. FMO3, along with several CYPs, is involved in THT oxidation to THT-1 oxide and probably the subsequent oxidation steps [168]. Accordingly, drugs metabolized via CYPs such as Cy are known to affect Bu plasma concentrations and kinetics [168–171].

GSH is also an important enzyme in 4-OH-Cy detoxification [42, 43]. As previously mentioned, Bu consumes up to 60% of hepatic GSH [172]. It is believed that the accumulation of the cytotoxic 4-OH-Cy due to GSH consumption by Bu may cause hepatic damage and increase the incidence of SOS [147] (Figure 3(b)). The time interval between the last Bu dose and the first Cy-dose is important in the development of SOS. A significantly lower incidence of SOS was found when this time interval was >24 hours compared to that seen when the interval was 12 hours [147, 160]. Furthermore, reversal of the administration order from Bu and then Cy to Cy and then Bu produced the same engraftment outcome but reduced the toxicity and improved the mortality of the conditioning regimen both in patients and mice [173–176]. Based on all of the above discussion, Bu/Cy conditioning protocols should be revised to take the drug administration sequence, and/or time interval between both drugs, into account in addition to considering the appropriate accompanying supportive therapy.

## 6. Opinion and Future Perspectives

In this review, we have summarized the most important studies investigating the effect of Cy pharmacogenomics on its kinetics. We have discussed the important older studies as well as the most recent research. Additionally, as well as focusing on CYP2B6, we have expanded our review to include the other important enzymes affecting Cy kinetics. We indicated the importance of enzymes such as CYP2C19, CYP2J2, POR, and GSTA1. Finally, we have discussed Bu/Cy conditioning as a clinical example of drug interactions that can be affected by the enzymes' polymorphisms.

Based on our review, it is evident that the CYP polymorphisms CYP2B6, CYP2C19, and POR change the Cy kinetics and hence change the clinical outcome. However, such an effect is complex and is clearly multifactorial. In some patients, the induction of other enzymes involved in Cy bioactivation may mask this effect.

Additionally, concomitant medications given during Cy treatment may induce (or suppress) particular CYPs and alter the Cy bioactivation. Such induction/suppression can be very variable and polymorphism dependent. Finally, a patient's age and clinical circumstances may play an important role in Cy's effect on the clinical outcome.

**Figure 4:**
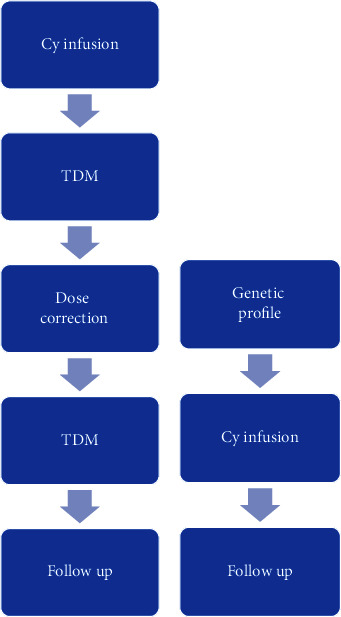
Cyclophosphamide personalized dose adjustment based on the currently available therapeutic drug monitoring (TDM) (a) and applying gene sequencing in the future (b).

Currently, the most commonly used method for personalized treatment is therapeutic drug monitoring (TDM) which starts after the patient's first dose. However, the application of TDM can be challenging because of its timing limitations, usually occurring late in the evening or at night. Additionally, TDM increases the work load on the nurses since they have to collect and process several blood samples after the end of the Cy infusion (Figure 4(a)). An alternative TDM strategy is a limited sampling model (LSM) which requires fewer blood samples to be taken (2–4 samples compared to 8–10 samples). However, it is still stressful to run the samples, obtain the results, then calculate the drug kinetics, and adjust the dose before the next infusion is required. Our review indicates that there is a lack of reliable LSMs for Cy kinetics calculations.

We believe that in the future, a patient's gene sequencing will be one of the routine investigations prior to the start of Cy treatment (Figure 4(b)). Gene sequencing results should be closely interpreted in order to understand the possible interactions between different enzymes' polymorphisms. Such investigations are of great importance, especially in some populations where specific polymorphisms are known to be common and can alter the Cy kinetics. Finally, accompanying treatment as well as Cy in combination with other cytostatics should be considered by the clinician in order to avoid drug interactions that may impact the clinical outcome. Examples of such scenarios are presented in Table 3. Taken together, we hope that this summary will help in facilitating personalized cyclophosphamide dose adjustment, reducing associated adverse side effects, and improving clinical outcomes.

## 7. Conclusion

Cyclophosphamide is a prodrug that is mainly bioactivated by CYP2B6. However, several other CYPs are involved in its bioactivation. The rate of bioactivation is controlled by the POR enzyme which can alter this step and markedly affect the drug kinetics. Additionally, other enzymes such as GSH/GST are also involved in the Cy metabolic pathway. The involvement of CYP2J2 in Cy metabolism can cause extrahepatic bioactivation and hence several side effects. The effect of any of the previous enzymes' polymorphisms on Cy kinetics and the clinical outcome have been extensively investigated; however, the picture here is still complex. In several studies, enzyme polymorphisms such as CYP2B6, CYP2C19, GSTA1, and POR were reported to alter the drug kinetics as well as the clinical outcome. However, other factors such as the patient's age, diagnosis, and clinical status could also alter Cy kinetics.

## Figures and Tables

**Table 1:** Common cyclophosphamide related drugs.

Alkylating agents share the cyclophosphamide mechanism of action	Common cyclophosphamide drug-drug interactions
Nitrogen mustards: cyclophosphamide, chlormethine, uramustine, melphalan, chlorambucil, ifosfamide, bendamustine	Adalimumab, baricitinib, BCG vaccine, bupivacaine, bupivacaine liposome, certolizumab, chloroprocaine, cidofovir, cladribine, clozapine, deferiprone, dengue vaccine, deucravacitinib, etanercept, etidocaine, fingolimod, golimumab, infliximab, influenza virus vaccine, inotersen, leflunomide, levobupivacaine, lidocaine, measles virus vaccine, mepivacaine, mumps virus vaccine, nadofaragene firadenovec, nalidixic acid, natalizumab, ozanimod, poliovirus vaccine, ponesimod, prilocaine, procaine, procaine penicillin, radium 223 dichloride, ritlecitinib, ropivacaine, rotavirus vaccine, rubella virus vaccine, siponimod, smallpox vaccine, strontium-89 chloride, talimogene laherparepvec, tenofovir, teriflunomide, tetracaine, thalidomide, thiotepa, tofacitinib, typhoid vaccine, ublituximab, upadacitinib, varicella virus vaccine, voclosporin, yellow fever vaccine, zoster vaccine
Nitrosoureas: carmustine, lomustine, streptozocin	
Alkyl sulfonates: busulphan, treosulfan	
Platinum: cisplatin, carboplatin, dicycloplatin, eptaplatin, lobaplatin, miriplatin, nedaplatin, oxaliplatin, picoplatin, satraplatin, triplatin tetranitrate	

**Table 2:** Summary of the enzymes involved in the cyclophosphamide metabolic pathway and the most common polymorphisms affecting its kinetics.

Enzyme family	Enzyme involved in cyclophosphamide metabolic pathway	Polymorphisms affecting cyclophosphamide kinetics	References
Aldehyde dehydrogenases (ALDH)	ALDH1A1	^*∗*^ *2, rs8187996*	Hassan et al. [112]
			Kalra et al. [60]
			Helsby et al. [57]
			Hwang et al. [110]
	ALDH3A1	^*∗*^ *2*	Afsar et al. [55]
			Ming et al. [96]

Cytochrome P450 (CYP)	CYP2B6	^*∗*^ *2,* ^*∗*^ *4,* ^*∗*^ *5,* ^*∗*^ *6,* ^*∗*^ *7,* ^*∗*^ *8,* ^*∗*^ *9*	Raccor et al. [54]
			Hassan et al. [112]
			Labib et al. [102]
			Shu et al. [101]
			Ming et al. [96]
			Helsby et al. [101]
	CYP2C9		Hassan et al. [112]
			Schirmer et al. [114]
	CYP2C19	^*∗*^ *2,* ^*∗*^ *17*	Afsar et al. [55]
			Jamieson et al. [108]
			Shu et al. [101]
			Kalra et al. [60]
			Ahmed et al. [58]
			Helsby et al. [101]
	CYP2D6		Hassan et al. [112]
			Cura et al. [153]
	CYP2J2	^*∗*^ *7, rs1056596*	El-Serafi et al. [115]
			Ahmed et al. [58]
	CYP3A4		Hassan et al. [112]
			Kumaraswami et al. [62]
	CYP3A5	^*∗*^ *3*	Hassan et al. [112]
			Kumaraswami et al. [62]
			Shu et al. [101]
			Ahmed et al. [58]

Glutathione S-transferase (GST)	GSTA1	^*∗*^ *B*	Afsar et al. [55]
			Hassan et al. [112]
	GSTP1	*rs1695*	Hassan et al. [112]
			Kumaraswami et al. [62]
			Attia et al. [154]
			Cura et al. [153]
			Hajdinak et al. [155]

Cytochrome P450 oxidoreductase (POR)	POR	^*∗*^ *28*	El-Serafi et al. [115]
			Ahmed et al. [58]

Italic values indicate a genetic polymorphism.

**Table 3:** Common examples of clinical scenarios that may occur during cyclophosphamide treatment.

Condition	Complication	Possible cause(s)	References
Cy infusion	High cy blood levels	CYP2B6 polymorphism	Hassan et al. [112]
			Shu et al. [101]
			Ming et al. [96]
			Helsby et al. [101]
		Drug-drug interactions	Hassan et al. [112]
			Conklin et al. [148]
			Cura et al. [153]
	Low cy blood levels	POR^*∗*^28 polymorphism	El-Serafi et al. [115]
			Ahmed et al. [58]
	Extrahepatic side effects	CYP2J2 polymorphism(s)	El-Serafi et al. [115]
			Ahmed et al. [58]

Bu-cy conditioning	High cy blood levels	GSH/CYPs consumption by bu	Hassan et al. [112]
			Kumaraswami et al. [62]
			Cura et al. [153]
			Hajdinák et al. [155]

Bu: busulphan; Cy: cyclophosphamide; CYP: cytochrome P450; GSH: glutathione; POR: cytochrome P450 oxidoreductase.

## Data Availability

No data were used to support the findings of this study.

## Additional Points

*What Is Already Known about This Subject*. Cyclophosphamide (Cy) is a prodrug that is activated predominantly by CYP2B6. There is a high interindividual variation in Cy kinetics that can be accounted for by CYP2B6 polymorphisms and/or other clinical factors. *What This Review Adds*. Enzyme polymorphisms alter Cy kinetics. However, such an effect is multifactorial as contributions from CYP2B6, other CYPs, and GSTs as well as POR polymorphisms may occur. The clinical factors (age, diagnosis, etc.) may mask the effect of these polymorphisms. Patient gene sequencing prior to the start of Cy treatment will certainly help in personalizing the most appropriate Cy dose and hence improve the clinical outcome.
